# Differential diagnosis and clinical predictors in suspected optic neuritis

**DOI:** 10.1007/s10384-025-01286-0

**Published:** 2025-10-23

**Authors:** Sotaro Mori, Kaori Ueda, Mari Sakamoto, Yuko Yamada-Nakanishi, Wataru Matsumiya, Akiko Miki, Hisanori Imai, Sentaro Kusuhara, Makoto Nakamura

**Affiliations:** 1 Division of Ophthalmology, Department of Surgery, Kobe University Graduate School of Medicine, Kobe, Japan; 2 Department of Ophthalmology, Kansai Medical University, Hirakata, Japan

**Keywords:** Optic neuritis, Differential diagnosis, Neuromyelitis optica spectrum disorders, Anterior ischemic optic neuropathy

## Abstract

**Purpose:**

Rapid diagnosis and initiation of treatment are essential to improve outcomes for optic neuritis. However, many patients suspected of having optic neuritis may have different underlying conditions. This study aimed to investigate the spectrum of diseases in patients referred with suspected optic neuritis and to identify clinical factors associated with confirmed optic neuritis.

**Study design:**

Retrospective cohort study

**Methods:**

This study retrospectively reviewed 255 cases referred to Kobe University Hospital with suspected optic neuritis between January 2016 and June 2024. Cases were excluded if patients had a history of optic neuritis, encephalitis, or myelitis, were referred from non-ophthalmology departments, or resided outside Hyogo Prefecture. Logistic regression analysis was conducted to identify factors significantly associated with confirmed optic neuritis.

**Results:**

Of the 206 eligible cases, 89 (43.2%) were confirmed to have optic neuritis. Other major diagnoses included anterior ischemic optic neuropathy (18.4%), space-occupying lesions such as intracranial tumors (11.7%), and retinal diseases or uveitis (10.2%). Logistic regression analysis revealed significant associations between confirmed optic neuritis and younger age, the presence of central scotoma, eye pain, decreased visual acuity, reduced critical flicker fusion frequency, and a shorter interval between symptom onset and consultation.

**Conclusion:**

Although 43.2% of suspected cases were confirmed as optic neuritis, a substantial proportion was attributed to other conditions requiring distinct diagnostic and therapeutic approaches. These findings emphasize the critical role of multidisciplinary collaboration and evidence-based protocols in managing patients with acute visual impairment.

**Supplementary Information:**

The online version contains supplementary material available at 10.1007/s10384-025-01286-0.

## Introduction

In recent years, the urgency to treat optic neuritis has grown significantly in the attempt to improve visual prognosis [1]. Traditionally, optic neuritis was considered a disease with a favorable prognosis by The Optic Neuritis Treatment Trial (ONTT) [2]. However, this outlook does not apply to neuromyelitis optica spectrum disorders (NMOSD), which account for 3–12% of optic neuritis cases [3, 4]. Optic neuritis associated with NMOSD is known to have a poor visual prognosis, but evidence indicates that early initiation of intravenous methylprednisolone therapy (IVMP) improves visual outcomes [5]. A recent review by the Neuromyelitis Optica Study Group (NEMOS) [6] highlighted retrospective studies in which 40% of patients achieved complete remission when treatment began within two days of onset, compared with only 3.7% when treatment was delayed beyond one week. These findings have led to a growing emphasis on expediting the treatment of optic neuritis. While ophthalmologists often diagnose the condition, its management is frequently overseen by neurologists [7], who advocate for the rapid transfer of suspected optic neuritis cases from ophthalmologists. Ophthalmologists generally support this proactive approach, as it benefits patient prognosis. However, not all cases of acute vision loss suspected to be optic neuritis are ultimately confirmed. For example, in infectious optic neuropathy, IVMP could be fatal [8]. Although neurologists may argue that cerebrospinal fluid analysis can exclude infectious causes before initiating steroids, many cases of suspected optic neuritis arise from non-infectious etiologies.

Diagnosing retrobulbar optic neuritis, which lacks optic disc swelling, can be particularly challenging. Acute vision loss often raises suspicion of optic neuritis, yet other causes are frequently implicated. Surprisingly, few studies have comprehensively analyzed the actual diagnoses in patients initially suspected of optic neuritis. An American study investigating 122 patients suspected of optic neuritis reports that only 49 (40.2%) were confirmed to have optic neuritis [9]. Notably, the study’s first author was a neurologist. In our clinical practice, we frequently encounter cases where patients are referred by internal medicine physicians for suspected optic neuritis due to eye pain. However, upon examination, many are found to have alternative diagnoses, such as eye pain from superficial punctate keratitis or unilateral visual loss without a relative afferent pupillary defect (RAPD), strongly suggestive of functional visual loss. Thus, patients whom ophthalmologists would not initially suspect to have optic neuritis are often referred as such.

In this study, we examined cases in which ophthalmologists initially suspected optic neuritis and analyzed the proportion of patients ultimately confirmed to have the condition. Whenever a substantial proportion of these patients are diagnosed with optic neuritis, it may justify bypassing detailed ophthalmological examinations at advanced institutions and referring patients directly to neurologists for prompt treatment. Additionally, we analyzed patient factors associated with optic neuritis to identify characteristics that may warrant early referral to neurologists.

## Methods

### Subjects

**Fig. 1:**
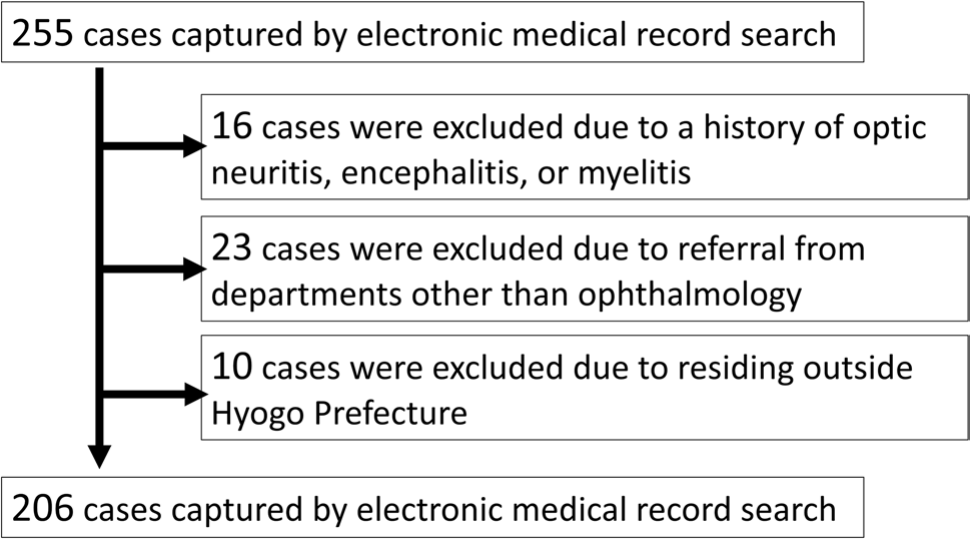
Flow diagram of patient selection

This study included all patients referred to Kobe University Hospital with suspected optic neuritis between January 2016 and June 2024. The following exclusion criteria were applied: (1) patients with a history of optic neuritis, encephalitis, or myelitis (16 cases); (2) cases referred from non-ophthalmology departments (23 cases); and (3) patients residing outside Hyogo Prefecture (10 cases). A flow diagram of patient selection is provided in Fig. 1.

For criterion (1), patients with a history of demyelinating diseases such as multiple sclerosis, NMOSD, or myelin oligodendrocyte glycoprotein antibody-associated disease (MOGAD) were excluded due to their inherently high likelihood of optic neuritis, which fell outside the scope. In this study within criterion (2), cases where optic neuritis was not strongly suspected—such as those presenting with eye pain unrelated to eye movement or without acute vision loss—were excluded. Many of these cases had alternative diagnoses, including central serous chorioretinopathy (CSC) or cataract, as detailed in Supplemental Table 1.

For criterion (3), referrals from outside Hyogo Prefecture were excluded because Kobe University Hospital’s neuro-ophthalmology expertise often attracts cases of acute vision loss from across Japan. Many of these cases have already undergone magnetic resonance imaging (MRI) evaluations at other hospitals, ruling out optic neuritis and identifying alternative diagnoses such as Leber’s hereditary optic neuropathy (LHON). Limiting the analysis to patients within Hyogo Prefecture ensured focus and consistency. Details of patients excluded under criterion (3) are provided in Supplemental Table 2.

### Primary outcome

The primary outcome was the proportion of patients with suspected optic neuritis who were ultimately confirmed to have the condition. Optic neuritis was diagnosed based on contrast-enhanced MRI findings of high signal intensity with optic nerve parenchyma enhancement. For two patients unable to undergo contrast-enhanced MRI due to asthma or severe renal dysfunction, diagnosis relied on high signal intensity of the optic nerve observed on short τ inversion recovery (STIR) images [10]. All patients diagnosed with optic neuritis underwent serum testing for anti-aquaporin-4 (AQP4) antibodies using the ELISA method. In this study, cell-based assay for AQP4 and testing for myelin oligodendrocyte glycoprotein (MOG) antibodies’ testing was not performed in all patients; MOG antibody testing was reserved for cases with clinical suspicion. Idiopathic optic neuritis was defined when both AQP4 and MOG antibodies were negative and no evidence of dissemination in time and space lesions suggestive of multiple sclerosis was observed.

In cases where MRI did not show optic nerve abnormalities but revealed a space-occupying lesion consistent with the patient's visual field defect, the etiology was classified as a space-occupying lesion. All patients in the study also underwent blood testing. If serological tests—Rapid Plasma Reagin (RPR), Treponema Pallidum Hemagglutination Assay (TPHA), or T-SPOT—were positive and exudative chorioretinitis was detected on fluorescein fundus angiography, the patient was diagnosed with syphilitic or tuberculous uveitis. Optical coherence tomography (OCT) was performed in all patients. When findings such as outer retinal layer atrophy suggested conditions including acute zonal occult outer retinopathy, autoimmune retinopathy, acute macular neuroretinopathy, or cancer-associated retinopathy, further evaluation with full-field or multifocal electroretinography was conducted. Final diagnoses of retinal and uveitic disorders were made by retina and uveitis specialists at our institution (W.M., A.M., H.I., S.K.).

Following MRI, blood tests, and fluorescein angiography, cases in which other diagnoses were excluded were evaluated by neuro-ophthalmologists (S.M., K.U., M.S., Y.Y.-N., M.N.) to determine the presence of ischemic optic neuropathy. Among these, anterior ischemic optic neuropathy (AION) was defined by the presence of optic disc swelling, and arteritic AION (A-AION) was further identified by an erythrocyte sedimentation rate (ESR) exceeding 50 mm/hour. Posterior ischemic optic neuropathy (PION), being a diagnosis of exclusion, was classified as unknown etiology and considered as suspected PION.

### Secondary outcome

Secondary outcomes included factors such as patient age, sex, symptom laterality (unilateral or bilateral), logMAR visual acuity, visual field constriction patterns, presence of optic disc swelling at the initial consultation, eye pain, critical flicker fusion frequency (CFF) at the first visit, and the number of days from symptom onset to hospital presentation. The Mann–Whitney *U* test was used to compare parameters between patients with confirmed optic neuritis and those diagnosed with other conditions. In addition, logistic regression analysis was performed to evaluate the association between these factors and the likelihood of an optic neuritis diagnosis.

### Evaluation parameters

Visual field constriction patterns were classified into five categories: central scotoma, altitudinal hemianopia, peripheral vision only, other constriction patterns, and normal [11]. In cases of severely impaired visual function, CFF and visual field measurements were not feasible; measurable cases are presented in the results table. The logMAR values for counting fingers, hand motion, light perception, and no light perception were assigned as 2.0, 2.4, 2.8, and 2.9, respectively, following a previous report [12].

### Ethics approval

This study adhered to the tenets of the Declaration of Helsinki and was approved by the Institutional Review Board of Kobe University (No. B240222). Consent to participate: Opt-out consent was obtained from all patients. This method was used for participant recruitment in the study. Informed consent was not obtained from the patients as this study was retrospective and observational. However, patients were allowed to withdraw their consent anytime in an opt-out fashion.

## Results

Table 1 and Supplemental Figure 1 summarize the final diagnoses of the 206 patients included in this study. Of these, 89 patients (43.2%) were confirmed to have optic neuritis. Among the remaining cases, the most frequent diagnoses were AION in 33 patients (16.0%), space-occupying lesions in 24 patients (11.7%), and retinal or uveitis-related diseases in 21 patients (10.2%). Among the eight patients with other optic neuropathies, three were diagnosed with LHON, two with varicella-zoster virus (VZV)-induced optic neuropathy, two with diabetic papillopathy, and one with papillitis. Of the 14 patients with unknown causes, seven had a history of dialysis or cerebral infarction, and posterior ischemic optic neuropathy was suspected based on their histories of acute vision loss. Of the 17 cases classified as other, five involved psychogenic visual disturbances; five were diagnosed with glaucoma, and three with pseudo-papilledema. Two cases were attributed to eye pain caused by migraine and eyestrain, while the remaining cases included superior segmental optic hypoplasia, malingering, amaurosis fugax, and unknown optic atrophy (one case each). Two glaucoma patients were initially referred for suspected optic neuritis due to central scotoma but were ultimately diagnosed with myopia-associated glaucoma. Notably, two glaucoma patients with a history of resolved high intraocular pressure (>30 mmHg) were initially misdiagnosed with optic neuritis. Similarly, pseudo-papilledema was often misidentified as optic neuritis due to optic disc swelling and concurrent visual decline from unrelated causes, such as keratitis. A malingering case presented with total blindness in one eye and a dilated pupil unresponsive to light, raising initial suspicion of optic neuritis. However, further investigation revealed a history of Munchausen syndrome. The patient, a physical therapist, had self-administered mydriatic eye drops acquired from the workplace.

**Table 1:** Detailed list of final diagnoses in patients suspected of optic neuritis

N = 206	
Optic Neuritis, 89	Idiopathic, 58: NMOSD, 14: MOGAD, 17
AION, 33	A-AION, 4: NA-AION, 29
Space Occupying Lesion, 24	Tumor, 14 (Metastatic, 7: Primary 7): Papilledema, 4: Rhinogenous, 3: Idiopathic Orbital Inflammatory Syndrome, 2: Hypertrophic Pachymeningitis, 1
Retinal Diseases or Uveitis, 21	AZOOR, 4: AIR, 3: AMN, 2: CAR 1: Syphilis, 3: Tuberculous, 2: VKH, 2: Sarcoidosis, 1: CRAO, 1: MEWDS, 1: Optic disc pit maculopathy, 1
Other Optic Neuropathy, 8	LHON, 3: VZV-induced 2: Diabetic Papillopathy, 2: Papillitis 1
Others, 17	Psychogenic Visual Disturbance, 5: glaucoma, 5: Pseudo-papilledema, 3: Eye Pain, 2: SSOH, 1: Malingering, 1: Amaurosis Fugax, 1: Unknown Optic Atrophy, 1
Unknown, 14	Suspected PION, 7

NMOSD, Neuromyelitis Optica Spectrum Disorders: MOGAD, Myeline Oligodendrocyte Glycoprotein Antibody Associated Disease: AION, anterior ischemic optic neuropathy: A-AION, Arteritic AION: NA-AION, Non-Arteritic AION: AIR, Autoimmune Retinopathy: AZOOR, Acute Zonal Occult Outer Retinopathy: AMN, Acute Macular Neuroretinopathy: CAR, Cancer Associated Retinopathy: VKH, Vogt-Koyanagi-Harada disease: CRAO, Central Retinal Artery Occlusion, MEWDS, Multiple Evanescent White Dot syndrome: LHON, Leber Hereditary Optic Neuropathy: VZV, Varicella Zoster Virus: SSOH, Superior Segmental Optic Hypoplasia: PION, Posterior Ischemic Optic Neuropathy

Table 2 presents the characteristics of patients categorized by disease type. In 89 patients diagnosed with optic neuritis, 14 (15.7%) tested positive for AQP4 antibodies (indicative of NMOSD), and 17 (19.1%) tested positive for MOG antibodies (indicative of MOGAD). Compared to patients with other diagnoses, those with optic neuritis were younger (median age 41 vs. 53 years, p < 0.0001), more likely to report eye pain (39.3% vs. 12.8%, p < 0.001), and more likely to exhibit central scotoma (47.2% vs. 18.0%, p < 0.001). While disc swelling was more frequent in optic neuritis patients, the difference was not statistically significant (61.8% vs. 48.7%, p = 0.07). In the 33 AION cases, all patients presented with disc swelling, and three of the four A-AION cases were bilateral at the initial presentation. Altitudinal hemianopia was observed in 18 cases (54.5%), often accompanied by relatively preserved visual acuity with a median logMAR of 0.3. In the 24 cases with space-occupying lesions, the median interval from symptom onset to hospital presentation was 16 days, with many patients presenting more than one month after onset.

**Table 2:** Characteristics of patients categorized by disease types

	All Patients (N = 206)	Optic Neuritis (N = 89)	AION (N = 33)	Space Occupying Lesion (N = 24)	Retinal Diseases or Uveitis (N = 21)
Type		Idiopathic, 58: NMOSD, 14: MOGAD, 17	A-AION, 4: NA-AION, 29	Tumor 14 (Metastatic, 7: Primary 7): Others, 10	AZOOR 4, AIR, 3: AMN, 2: Syphilis, 3: Others, 9
Sex	Male, 86: Female, 120	Male, 37: Female, 52	Male, 16: Female,17	Male, 6: Female, 18	Male, 9: Female, 12
Age, y.o.	53 (34, 68)	41 (29, 57)	67 (61, 76)	67 (53, 74)	55 (38, 74)
Right or Left	Right, 91: Left, 90: Both, 25	Right, 41: Left, 41: Both, 7	Right, 17: Left, 13: Both, 3	Right, 10: Left, 9: Both, 35	Right, 12: Left, 7: Both, 2
logMAR	0.9 (0.2, 1.7)	1.3 (0.7, 2.3)	0.3 (0, 1.2)	0.8 (0, 1.8)	0.4 (0, 1.0)
Days from Onset to Visit	7 (3, 14)	5 (3, 10)	7 (3, 10)	16 (7, 35)	9 (3, 30)
Disc Swelling	Yes, 94: No, 112	Yes, 34: No, 55	Yes, 33: No,0	Yes, 12: No, 12	Yes, 11: No, 10
Eye Pain	Yes,50: No, 156	Yes, 35: No, 54	Yes, 3: No, 30	Yes, 2: No, 22	Yes,1: No, 20
Critical Flicker Frequency, Hz	26 (16, 37)	16 (12, 26)	27 (22, 37)	27 (22, 37)	31 (23, 39)
Visual Field Test	CS, 63: AH, 36, Peri,20: Others, 41: Normal, 18: Unmeasurable, 28	CS, 42: AH, 11: Peri, 11: Others, 5: Normal, 1: Unmeasurable, 19	CS, 3: AH, 18: Peri, 3: Others, 6: Normal, 0: Unmeasurable, 3	CS, 3: AH, 1: Peri, 4: Others, 10: Normal, 3: Unmeasurable, 3	CS, 6: AH, 3: Peri, 0: Others, 11: Normal, 0: Unmeasurable, 1

NMOSD, Neuromyelitis Optica Spectrum Disorders: MOGAD, Myeline Oligodendrocyte Glycoprotein Antibody Associated Disease: AION, anterior ischemic optic neuropathy: A-AION, Arteritic AION: NA-AION, Non-Arteritic AION: AZOOR, Acute Zonal Occult Outer Retinopathy: AIR, Autoimmune Retinopathy: AMN, Acute Macular Neuroretinopathy: CS, Central Scotoma: AH, Altitudinal Hemianopsia: Peri, Peripheral vision only. Continuous variables were shown as medians (interquartile range) and categorical variables were shown as number

Table 3 presents logistic regression analysis results identifying factors significantly associated with a diagnosis of optic neuritis. Younger age, central scotoma, eye pain, decreased visual acuity, reduced critical flicker fusion frequency (CFF), and a shorter interval from onset to consultation were significant predictors. In contrast, gender, bilaterality, and optic disc swelling were not significant predictors. Positive predictive values for each factor are provided in Table 4.

**Table 3:** Multiple regression analysis of factors associated with optic neuritis

	Odds Ratio	95% Confidence interval of Odds Ratio	*p* value
Age	0.96	0.95, 0.98	**<0.001**
Sex to male	1.14	0.53, 2.45	0.74
Bilateral	0.43	0.12, 1.56	0.20
Central Scotoma	3.16	1.38, 7.26	**<0.01**
Disc Swelling	0.58	0.26, 1.27	0.17
Eye Pain	3.58	1.38, 9.27	**<0.01**
logMAR	1.72	1.07, 2.76	**0.03**
Critical Flicker Frequency Decrease (<30 Hz)	0.18	0.06, 0.57	**<0.01**
Days from Onset to Visit	0.96	0.93, 0.99	**0.01**

**Table 4:** Positive predictive value of each parameter for optic neuritis

Young Age (< 40 y.o.)	59.7%
Female	43.3%
Bilateral	28.0%
Central Scotoma	66.7%
Disc Swelling	53.1%
Eye Pain	70.0%
Critical Flicker Frequency Decrease (<30 Hz)	57.0%
Days from Onset to Visit (<8 days)	52.7%

## Discussion

This study assessed the proportion of cases referred by ophthalmologists as suspected optic neuritis that were ultimately confirmed to have the condition. Notably, even when referrals were made by ophthalmologists, the confirmation rate for optic neuritis was relatively low at 43.2%. Among these, 14 patients (6.8%) were diagnosed with NMOSD, highlighting the critical role of initial ophthalmological evaluation prior to referral to a neurologist.

A report from Japan investigated the causes of optic disc swelling [13]. In that study of 93 patients with optic disc swelling, 60 had unilateral and 33 had bilateral swelling; among them, 21 were diagnosed with AION and 20 with optic neuritis. In our cohort of patients with suspected optic neuritis, 94 (45.6%) presented with disc swelling, of which 83 (88.3%) were unilateral and 11 (11.7%) were bilateral. The previous report also included cases with small optic discs and other conditions, which may explain differences in distribution. Notably, among our 94 patients with disc swelling, 34 (36.2%) were diagnosed with optic neuritis and 33 (35.1%) with AION—percentages comparable to the previous study.

Our findings suggest that patients suspected of having optic neuritis are more likely to be diagnosed with the condition when multiple significant clinical factors, as shown in Tables 3 and 4, are present. However, these clinical features alone are insufficient for a definitive diagnosis. Comprehensive evaluation—including MRI—is ultimately required in patients suspected of optic neuritis. The results of this study may help guide clinical decision-making by indicating when immediate MRI is warranted versus when alternative tests, such as blood sampling or fundus fluorescein angiography, should be prioritized.

Interestingly, retinal diseases and uveitis—conditions not typically considered neuro-ophthalmological in nature—accounted for 10.2% of cases. These diagnoses were often based on macular oedema or outer retinal abnormalities identified through OCT [14, 15]. Neuro-ophthalmologists should remain vigilant for such findings during evaluations, as they may provide insights pertinent for the underlying diagnosis.

The most commonly misdiagnosed condition was AION. All patients ultimately diagnosed with AION had optic neuritis excluded by MRI. Our results indicate that age, optic disc swelling, presence or absence of eye pain, and specific visual field defect patterns are helpful in distinguishing optic neuritis from AION. In particular, elderly patients presenting with disc swelling, no eye pain, and horizontal hemianopia are more likely to have AION. The age distribution at onset for optic neuritis and AION is already known to differ significantly [16], and it is important to recognize this distinction during clinical evaluation. However, distinguishing AION from NMOSD, which can have severe consequences if misdiagnosed, remains particularly challenging. Although NMOSD frequently affects older patients [3] and may present with altitudinal hemianopia [17], relying solely on age or visual field patterns for differentiation is insufficient. Diagnostic confirmation requires MRI, blood tests, and fluorescein angiography when AION is strongly suspected.

Contrary to common assumptions, optic disc swelling and gender were not definitive indicators of optic neuritis in this study. While optic disc swelling was observed in 61.8% of patients with optic neuritis (55 out of 89), it was universally present in AION cases and frequently observed in uveitis patients. This likely contributed to its lack of statistical significance as a diagnostic factor. Similarly, although optic neuritis is generally more prevalent in women, gender did not emerge as a significant predictor. This may be attributed to the inclusion of other conditions, such as meningiomas, which are more common in women. For example, data from the Central Brain Tumor Registry of the United States (CBTRUS) indicate that women are 2.3 times more likely than men to develop meningiomas [18]. The study also revealed that a longer interval between symptom onset and consultation reduced the likelihood of an optic neuritis diagnosis. While optic neuritis typically presents with acute visual impairment, some cases exhibit a subacute course characterized by initial improvement followed by deterioration [19]. However, these cases are often attributable to space-occupying lesions, such as intracranial or rhinogenic optic neuropathy, rather than optic neuritis.

This study has several limitations. As it was conducted at a single institution, regional biases may have influenced the findings. For example, Japan is the country with the most widespread MRI use in the world. Therefore, it is conceivable that in other countries with different medical facilities, a different process is required to diagnose optic neuritis. In addition, this study was limited to Hyogo Prefecture in Japan. For instance, the observed prevalence of Leber’s hereditary optic neuropathy—a hereditary condition—may reflect regional genetic or referral patterns. In addition, because this study was based on referrals from ophthalmologists, the proportion of patients with optic neuritis who also had demyelinating diseases such as multiple sclerosis or neuromyelitis optica may have been underestimated. Additionally, this study focused on patients referred for suspected optic neuritis, potentially underestimating the prevalence of retinal diseases identified through OCT at the initial clinic. Similarly, pituitary adenomas, which commonly cause intracranial space-occupying lesions [20], were not identified in this cohort. This may be because these lesions typically present with bitemporal hemianopia [21], a feature not commonly mistaken for optic neuritis.

In conclusion, the rate of confirmed optic neuritis among referred cases was 43.2%, with 14 patients (6.8%) diagnosed with NMOSD, a condition requiring prompt treatment. Given the wide range of differential diagnoses, ophthalmologists must conduct comprehensive evaluations—including OCT, MRI, blood tests, and fluorescein angiography—to rapidly and accurately identify the underlying condition. Young patients with acute-onset central scotoma, reduced visual acuity, and CFF should undergo an urgent MRI to facilitate prompt neurology consultation.

## Supplementary Information

Below is the link to the electronic supplementary material.

Supplementary file1 (DOCX 21 kb)

Supplemental Figure 1. Disease type in Patients Suspected of Optic Neuritis (TIF 806 kb)
