# Supplementary material for: Differential diagnosis and clinical predictors in suspected optic neuritis
Source: Jpn J Ophthalmol. 2025 Oct 23;70(2):367–73. doi: 10.1007/s10384-025-01286-0 (PMC13091875; doi:10.1007/s10384-025-01286-0)
Supplement: Supplementary file 1 — Supplementary file1 (DOCX 21 kb) [file 10384_2025_1286_MOESM1_ESM.docx]

**Supplemental Table 1.** Detailed List of Cases Referred from Non-Ophthalmology Departments

| (N=23) | Those with a history of demyelinating disease, 11 |
| --- | --- |
|  | NA-AION (Already diagnosed at another ophthalmology clinic), 2 |
|  | Eye Pain (Keratitis), 2: Cataract, 2, Secondary Cataract, 1 |
|  | CSC, 1: BRVO, 1 |
|  | Scleritis, 1: Floaters, 1: Presbyopia, 1 |

NA-AION, Non-Arteritic Anterior Ischemic Optic Neuropathy: CSC, Central Serous Chorioretinopathy: BRVO, Branch Retinal Vein Occlusion

**Supplemental Table 2.** Detailed List of Cases Residing Outside Hyogo Prefecture

| (N=10) | LHON, 4 |
| --- | --- |
|  | Unknown Optic Atrophy, 4 |
|  | Rhinogenous, 1: AIR, 1 |

LHON, Leber Hereditary Optic Neuropathy: AIR, Autoimmune Retinopathy
