# Supplementary figures and images for: Differential diagnosis and clinical predictors in suspected optic neuritis
Source: Jpn J Ophthalmol. 2025 Oct 23;70(2):367–73. doi: 10.1007/s10384-025-01286-0 (PMC13091875; doi:10.1007/s10384-025-01286-0)

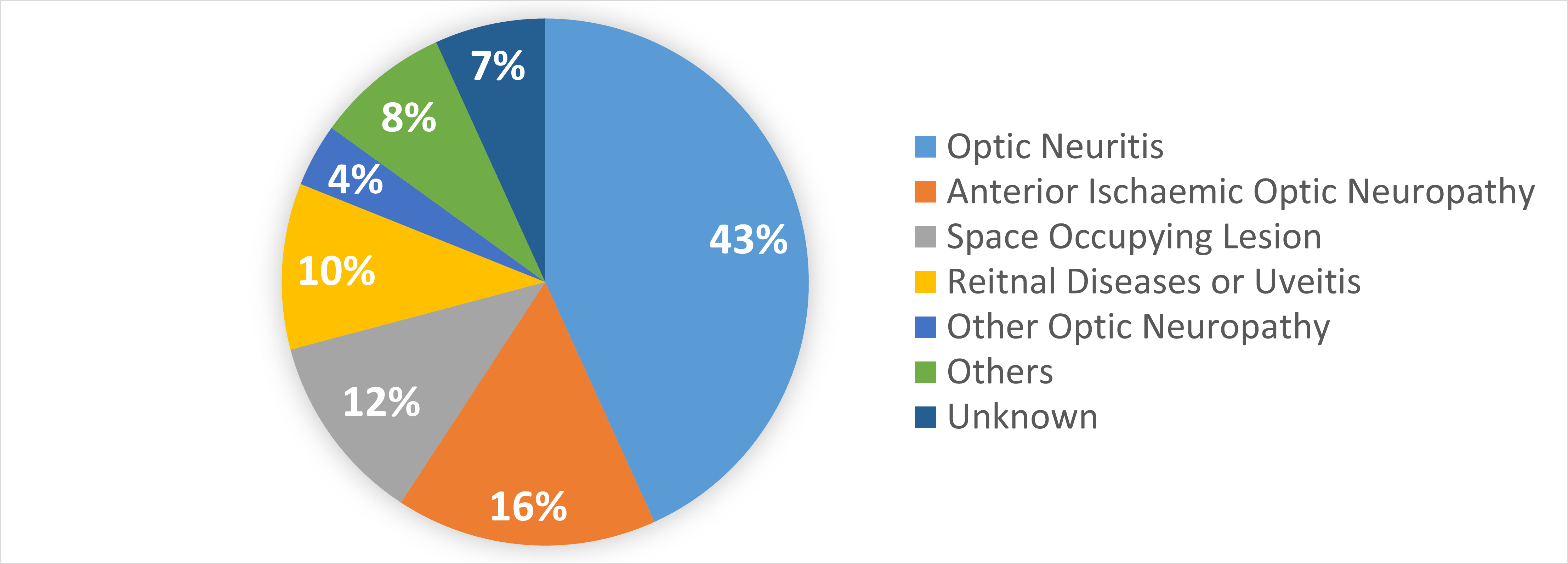

Supplement: Supplementary file 2 — Supplemental Figure 1. Disease type in Patients Suspected of Optic Neuritis (TIF 806 kb) [file 10384_2025_1286_MOESM2_ESM.tif]
